# P018 Reintroducing Burosumab in Symptomatic Adult X-Linked Hypophosphataemic Rickets

**DOI:** 10.1093/rap/rkag113.048

**Published:** 2026-11-03

**Authors:** Mays Ajeel Jabbar Al-Sarraji, Chadi Rakieh

**Affiliations:** The Robert Jones and Agnes Hunt orthopaedic hospital, Oswestry, United Kingdom; The Robert Jones and Agnes Hunt orthopaedic hospital, Oswestry, United Kingdom

## Abstract

**Introduction:**

X-linked hypophosphataemic rickets (XLH) is a rare inherited phosphate-wasting disorder caused by pathogenic variants in the *PHEX* gene. Burosumab has transformed the management of children with XLH and is now approved for symptomatic adults. However, evidence regarding patients who discontinue treatment at final height and subsequently require re-initiation in adulthood remains limited. We present a young adult with persistent skeletal complications despite previous therapy, highlighting the ongoing burden of disease and the rationale for restarting burosumab.

**Case description:**

A young man with genetically confirmed XLH due to a *PHEX* pathogenic variant was diagnosed at 2½ years of age following investigation of bowed legs. There was no family history. He received conventional treatment with phosphate supplements and alfacalcidol before commencing burosumab in January 2021, which was discontinued in August 2023 after reaching final height. Following discontinuation, he resumed alfacalcidol 3 micrograms daily with phosphate supplementation, although adherence to phosphate was poor because of diarrhoea.

He re-presented with progressive pain affecting the knees, shins and ankles, associated with stiffness, mainly after period of inactivity and reduced mobility. He had been unable to continue playing rugby because of pain. His history included bilateral lower limb deformity, previous bilateral eight-plate surgery, Osgood-Schlatter disease, recurrent dental abscesses with loss of several teeth, and stable bilateral medullary nephrocalcinosis.

Examination demonstrated persistent lower limb deformity without synovitis or restriction of joint movement. Bone mineral density was normal for age (lumbar spine Z-score +2.8; femoral neck Z-score +2.2). Biochemical assessment and renal monitoring were undertaken, and repeat renal ultrasound was arranged.

Given persistent symptomatic disease and eligibility under NICE guidance, burosumab was recommenced with discontinuation of alfacalcidol at treatment initiation. Funding was requested, and follow-up was planned after six months of therapy.

**Discussion:**

Although skeletal growth ceases in adulthood, XLH remains a lifelong disorder associated with pain, stiffness, impaired physical function, enthesopathy, dental complications and reduced quality of life. This case illustrates that significant symptomatic disease may persist despite previous treatment during adolescence, particularly when therapy is initiated relatively late.

The patient’s normal bone mineral density highlights that dual-energy X-ray absorptiometry may not reflect disease severity or symptom burden in XLH. Clinical assessment should instead focus on pain, function, mobility and extraskeletal manifestations.

Adult eligibility for burosumab offers an opportunity to improve phosphate homeostasis and address ongoing symptoms in selected patients. This case also highlights practical challenges with conventional therapy, including poor adherence to oral phosphate because of gastrointestinal adverse effects, which may contribute to persistent disease activity. Careful renal surveillance remains important because of pre-existing nephrocalcinosis.

Re-initiation of burosumab following discontinuation at final height represents an evolving area of clinical practice and reinforces the importance of lifelong follow-up for adults with XLH within specialist metabolic bone services.

**Key learning points:**

• XLH is a lifelong disorder requiring continued specialist follow-up beyond attainment of final height.

• Adult patients may continue to experience significant pain, stiffness and functional limitation despite previous treatment during childhood or adolescence.

• Normal bone mineral density does not exclude clinically significant disease in XLH.

• Poor adherence to conventional phosphate therapy because of gastrointestinal side effects is common and may limit treatment effectiveness.

• Adults meeting NICE eligibility criteria may benefit from burosumab, including re-initiation after previous discontinuation at final height.

• Ongoing monitoring should include assessment of symptoms, physical function, dental health and renal complications such as nephrocalcinosis.

